# A 37 years [1984–2021] Landsat/Sentinel-2 derived snow cover time-series for Switzerland

**DOI:** 10.1038/s41597-025-04961-6

**Published:** 2025-04-15

**Authors:** Charlotte Poussin, Pascal Peduzzi, Bruno Chatenoux, Gregory Giuliani

**Affiliations:** 1 University of Geneva, Institute for Environmental Sciences, GRID-Geneva, Bd. Carl-Vogt 66, Geneva, CH-1211 Switzerland; 2 University of Geneva, Institute for Environmental Sciences, enviroSPACE Lab., Bd. Carl-Vogt 66, Geneva, CH-1211 Switzerland

**Keywords:** Environmental impact, Cryospheric science

## Abstract

Switzerland, renowned for its mountainous landscapes, holds nearly 10% of Europe’s water reserves, with 40% of its running waters originating from snowmelt. Snow plays a crucial role in the country’s water management, hydroelectric power, and alpine ecosystems. It supports freshwater supply, agriculture, and tourism, making accurate snow monitoring vital for resource management and environmental preservation. Climate change, however, threatens snow cover, impacting water availability, biodiversity, and ecosystem services. Remote sensing technologies have emerged as key tools for monitoring snow cover, providing critical data for climate models, hazard prediction, and resource planning. In Switzerland, snow cover is monitored using ground-based measurements, remote sensing, and climate models, with datasets from satellites like Landsat and Sentinel-2 offering valuable insights despite challenges such as cloud obstruction. Such data are essential for hydrological modelling, agricultural monitoring, and climate studies, contributing to our understanding of global warming and aiding in natural hazard assessment. Hereafter, we present a 37-year monthly time-series of snow cover derived from Landsat and Sentinel-2 data using the Snow Observations from Space algorithm and processed in the Swiss Data Cube that facilitates the analysis, production and reuse of this Essential Climate Variable, enhancing environmental monitoring efforts at national scale.

## Background & Summary

Switzerland is internationally known for its snow-covered mountainous landscapes and recognized as the “water castle of Europe” having almost 10% of Europe’s water reserves^1^. Among these reserves, 40% of running waters come from snowmelt^1–3^, making snow a critical element of the water cycle for both Switzerland and the European region. Snow plays a critical role in water resources management, hydroelectric power generation, and the alpine ecosystem. Furthermore, snow-covered mountains attract tourists for winter sports, contributing significantly to the Swiss economy^4^. Snow also influences local climate conditions, affecting agriculture and natural habitats. Consequently, accurate monitoring of snow cover is vital for managing water resources, ensuring energy security, supporting economic activities, and preserving environmental health in Switzerland^5–7^.

Mountainous environments are particularly sensitive to climate change^8^, with studies showing that warming is more pronounced at higher elevations, resulting in glacier retreat, reductions in the extent, quantity, and duration of snow cover, and the thawing of permafrost^5^. Snow cover is also critical for understanding and predicting various environmental and climatic processes^7^. Its high albedo reflects a large portion of solar radiation back into space, influencing Earth’s energy balance. Mountainous environments are particularly sensitive to climate change^8^, with studies showing that warming is more pronounced at higher elevations. This results in glacier retreat, reduced snow cover, and thawing permafrost^5^, with profound implications for water availability, biodiversity, and ecosystem functions^9^. According to recent climate scenarios^10^ for Switzerland, the proportion of water provided by snowmelt will decrease significantly^1^, affecting major rivers such as Rhône, Rhine and Danube. Therefore, continuous monitoring of snow-related parameters is crucial to support and develop efficient and effective mitigation and adaptation measures to address environmental changes^11^.

Snow cover is fundamental for understanding and predicting various environmental and climatic processes^7^. It influences Earth’s energy balance due to its high albedo, which reflects a large portion of solar radiation back into space. Monitoring snow cover is also essential for predicting natural hazards such as avalanches and floods.

Snow cover is recognized by the Global Climate Observing System (GCOS) as an Essential Climate Variable (ECV)^12^. As an ECV, snow cover provides critical data for understanding and predicting climate dynamics.

In Switzerland, snow is rigorously monitored using a combination of ground-based measurements, remote sensing technologies, and climate models^13^.

Among these different techniques, remote sensing technologies has gained a lot of interest over the past decades and has demonstrated to be a suitable source to complement scattered *in-situ* measurements^14^. The unique spectral signature of snow allows distinguishing it from other land cover classes. Algorithms such as the Normalized Difference Snow Index (NDSI) or more advanced techniques based on machine learning could be applied on Analysis Ready Data (ARD) to classify and map snow cover and quantify snow extent, depth, and water equivalent^15,16^. By providing timely and precise snow cover data, remote sensing enhances our understanding of snow dynamics and supports informed decision-making in response to climatic and environmental changes^4^.

Currently, there are various sources providing time-series data of snow cover in Switzerland derived from satellite observations such as MODIS that has been used to study the inter-annual variations of snow days across different climatic regions in Switzerland^17^. ESA Climate Change Initiative (CCI) Snow Cover provides long-term snow cover data. This includes snow extent and snow water equivalent data, which can be useful for understanding snow cover dynamics in Switzerland over several decades^18,19^. AVHRR data has also produced the longest snow cover time-series^20–22^. Although these datasets provide valuable information with high temporal resolution (e.g., daily) they suffer from coarse spatial resolution (ranging from 300 m to 1 km), which limits their applicability in small countries with a diversity of landscapes such as Switzerland^15,22^. Indeed, finer resolution is crucial for accurately representing snow cover dynamics in mountainous regions like Switzerland, where topographic variability occurs over relatively small areas, and snow cover properties can vary at scales of 10–100 m. Additionally, long-term snow cover time series (spanning over 30 years) are essential for studying global or regional snow cover changes in the context of climate change^20^ and for separating long-term trends from natural climate variability^23^. In recent year, new snow-related products based on Landsat and Sentinel offering higher spatial resolution (respectively 30 and 10 m) but with slightly lower temporal resolution (16 and 5 days) have emerged but not routinely generated for Switzerland^15,24,25^. The synergetic use of this virtual constellation of sensors could potentially produce information every 3 days^26–29^, dating back to the 1980s, making these sensors promising but also challenging due to the potential obstruction of clouds. Over the past two decades, various methods have been developed to enhance snow cover mapping, including cloud removal techniques such as multi-sensor analyses and the application of temporal and spatial filters^30–32^. Building on these advancements, this dataset incorporates advanced methodologies to mitigate cloud cover obstruction, enhance snow cover detection, and leverage the high spatial resolution and long-term records of Landsat and Sentinel-2 data, resulting in a more consistent and reliable snow cover product. Switzerland’s complete satellite archive (Landsat Collection 1 and Sentinel-2) in Analysis Ready Data format within the Swiss Data Cube (SDC – http://www.swissdatacube.ch) provides the foundation for generating this dataset. The objective is to deliver a medium to high-spatial resolution, consistent monthly snow cover time series for the entire country. This dataset is valuable for diverse applications, including hydrological modelling to predict water availability, streamflow, and reservoir levels critical for water management and planning^33^. It also supports agricultural monitoring by assessing snowmelt impacts on soil moisture, crop cycles, and ecological studies^34^ by tracking habitats conditions for wildlife and conserving biodiversity. Furthermore, snow cover dataset contributes to climate research by providing insights into seasonal and long-term cryosphere changes, enhancing our understanding of global warming trends and feedback mechanisms^35^. It plays a role in natural hazard assessments, helping predict and mitigate the impacts of floods and avalanches^36^ as well as helping archaeologists to identify potential areas of conservation and protection of archaeological remains^37^. The interoperability and scalability of snow cover data, facilitated by advancements in remote sensing and data integration technologies, increase its value, allowing it to be seamlessly incorporated into diverse environmental monitoring frameworks^38^.

## Methods

**Fig. 1:**
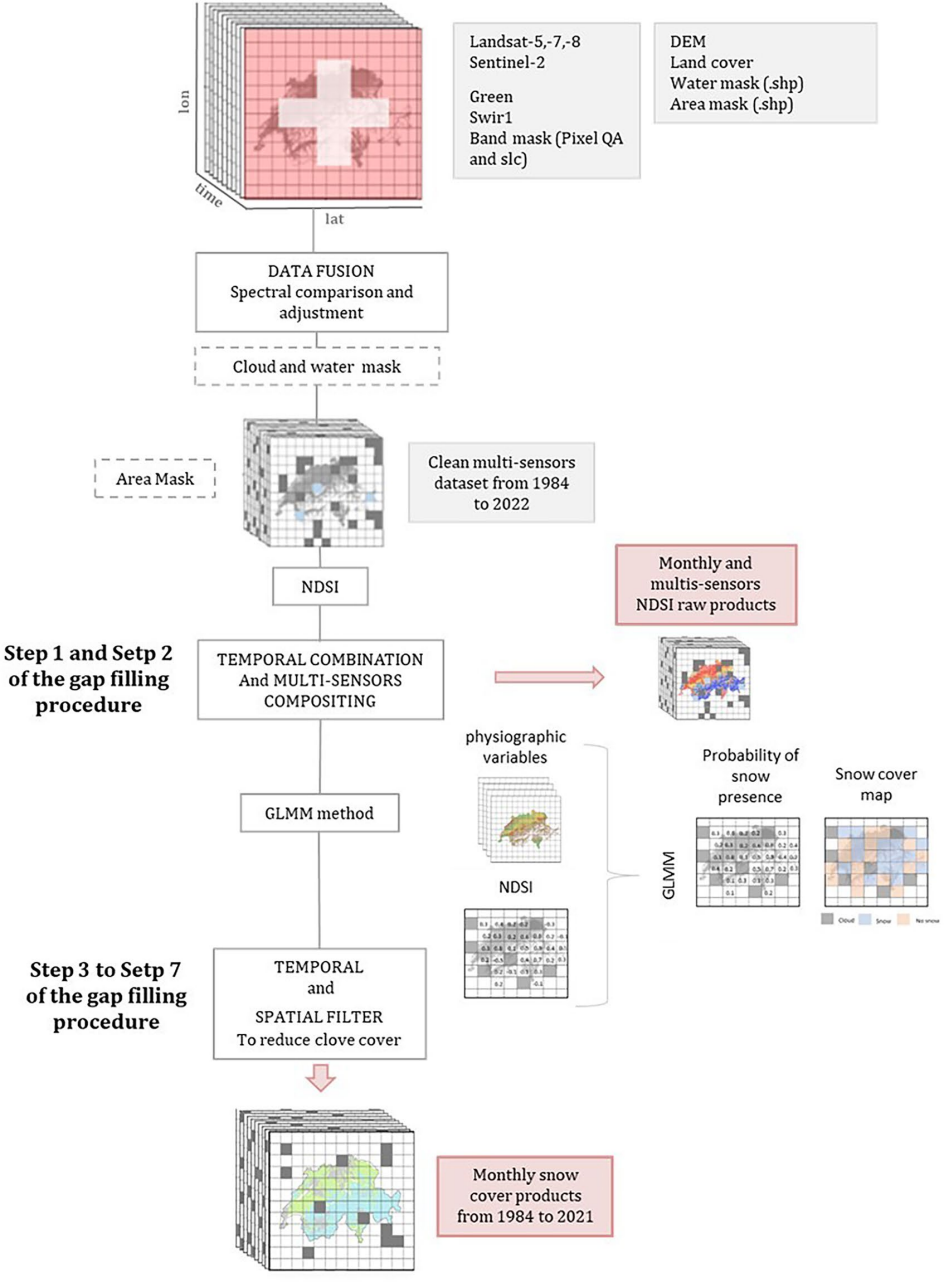
Snow Observation from Space (SOfS) processing workflow.

The development of this dataset followed 4 main steps as implemented in the Snow Observations from Space (SOfS) algorithm (Fig. 1): (1) satellite data provision, (2) Snow Observation from Space, (3) data validation, and (4) data publication in SwissEnvEO.

### Satellite data provision

Switzerland has developed a satellite Earth Observation (EO) ARD archive encompassing the entire national territory. The Swiss Data Cube (SDC – http://www.swissdatacube.ch), is a tera-scale analytical cloud-based platform that enables users to access, analyze, and visualize over 40 years of optical (e.g., Sentinel-2; Landsat-5, -7, -8, -9) and radar (e.g., Sentinel-1) EO data, starting from 1984^39,40^. The SDC supports national-scale analyses of large volumes of consistently calibrated satellite EO data. It aims to contribute to the government’s environmental and reporting mandates while providing Swiss research institutions with valuable satellite EO data^41–43^. The SDC has been used to assess the impact of climate change on vegetation^44,45^, snow^46^, threatened species^47^, as well as modelling species distribution^48^ or monitoring Sustainable Development Goals (SDG)^49,50^. The archive, updated daily, contains approximately 80,000 scenes, totalling 30 TB and over 3,000 billion observations/pixels. Recently, additional official raster datasets from the Swiss government, such as Land Use Statistics, Digital Elevation Model, and climate model outputs, have been included. The SDC is built on the Open Data Cube (ODC) software suite, an open-source project initiated by Geoscience Australia, the Commonwealth Scientific and Industrial Research Organization (CSIRO), the USGS, NASA, and the Committee on Earth Observations Satellites (CEOS)^51^. The ODC provides a framework for accessing, storing, managing, and analyzing large quantities of gridded satellite EO data collections. It features a Python-based Application Programming Interface (API) for data analysis and enables the cataloguing of extensive satellite EO data, ensuring data provenance tracking for quality control and updates^52^.

### Snow Observations from Space

The SOfS algorithm has been originally designed and implemented in the SDC by Frau *et al*.^53^ and further refined by Poussin *et al*.^54^. This final version encompasses various processing steps to maximize the detection of snow.

#### Cloud and water masking

Observations from optical satellites can be degraded by factors like instrument failure or cloud interference^55,56^. We filtered the optical data in the SDC using cloud mask information. For Landsat, cloud cover was estimated by the Collection 1 Level-1 QA 16-bit Band, using the CFMask algorithm^57^. Pixels labelled “clear,” “water,” or “snow” were considered clear, while other QA band attributes were marked as “cloud” and reanalyzed during gap filling. For Sentinel-2, the Sen2Cor correction algorithm flagged clouds and shadows^56^. Pixels labelled “vegetation,” “not vegetated,” “water,” “unclassified,” and “snow” were considered clear, with the remaining categories classified as “cloud” and reanalyzed during gap filling to increase clear observations. The percentage of clear observations per pixel over the entire time series can be determined using the following equation:

$${P}_{\left(x,y\right)}^{{Quality}}\left( \% \right)=\frac{\sum ({P}_{\left(x,y,t\right)}={\rm{clear\; observations}})}{{N}_{\left(x,y\right)}}\ast 100$$

Where *x,y* are the latitude, longitude respectively of the pixel P and *Nx,y* is the total number of scenes covering the pixel *x,y* in the period 1984–2021.

#### Snow cover detection

Once data is filtered to obtain clear observations, the NDSI has been computed for more than 4030 Landsat and Sentinel-2 scenes over the period 1984–2021. NDSI is defined as the reflectance difference between visible (green) and shortwave infrared (SWIR) wavelengths and is calculated with the following formula^58–60^:

$${NDSI}=({R}_{{green}}-{R}_{{SWIR}})/({R}_{{green}}+{R}_{{SWIR}})$$

Where R_green_ and R_SWIR_ are the surface reflectance in the green and in the SWIR bands. The index ranges from −1 to +1. Raw NDSI data were integrated into the binomial generalized linear mixed model (GLMM) by Poussin *et al*.^54^ to model the relationship between snow presence and NDSI. This approach incorporates elevation, land cover type, and seasonality to improve snow cover mapping accuracy compared to a fixed NDSI threshold, reducing omission errors while keeping commission errors low. It significantly enhances sensitivity while maintaining excellent specificity, enabling snow detection at lower NDSI values and minimizing false positives. Moreover, the model accounts for the complexity of the snow-NDSI relationship, offering a more robust and contextually appropriate solution for snow detection in Switzerland. To generate binary snow cover maps, we applied a probability threshold that optimizes the True Skill Statistic (TSS) index, classifying pixels as:

- 0: snow-free (land)
- 1: covered with snow
- 2: covered with clouds (including shadows)
- NA: water or outside Switzerland

#### Gap filling procedure

**Fig. 2:**
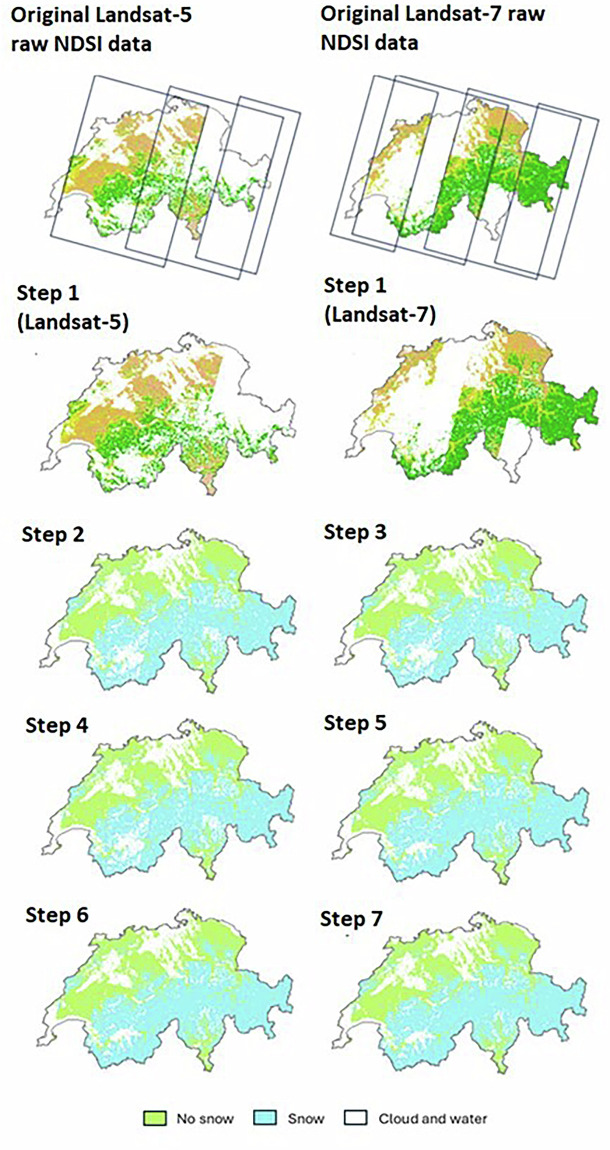
Cloud cover reduction process for Landsat-5 and Landsat-7 NDSI data during December 2000, showing the progression through seven steps. The original Landsat-5 NDSI data consists of three images, while the Landsat-7 NDSI data consists of six images. Step 1 represents the raw NDSI data, while steps 2 to 7 show binary snow cover products. Snow is represented in light blue, no snow (land) in light green, and clouds and lakes in white. The final snow cover product is depicted after step 7.

To further reduce cloud cover influence, the SOfS algorithm uses spatial and temporal techniques in a seven-step gap filling procedure. Each step incrementally removes a portion of cloudy pixels, with the resulting cloud-reduced product serving as the input for the next step. Successive iterations progressively reduce cloud presence in the snow cover products, though complete elimination of clouds is not achieved. As an example, Fig. 2 illustrates the progressive reduction in cloud cover fraction after each step for December 2000. Using this seven-step methodology, approximately 73% of cloud cover was removed for the example of December 2000, leaving less than 17% of pixels affected by clouds in this specific case (Fig. 2 – Step 7).

### Step 1 - Temporal combination of NDSI

First, raw NDSI data are combined into monthly composites using the maximum NDSI value per pixel for each image. Cloud cover is only reported when a given pixel is covered by clouds for the entire time frame. This technique removed 31% of cloud-obscured pixels in monthly composites with an average of 60% of clear observations per monthly product, regardless of the satellites.

### Step 2 - Multi-sensors compositing

The second step combines data from different sensors with slightly offset acquisition times to account for varying cloud coverage during satellite observations over Switzerland. By leveraging this temporal overlap, the likelihood of obtaining cloud-free observations is increased. The Landsat long-term data record, enhanced by the Sentinel-2 constellation’s higher spatial and temporal resolutions^61^, allows for combined datasets suitable for long-term high-frequency monitoring^62^. This combination reduces the global average revisit interval to 4.6 days^27^, creating denser time series for monitoring changes^63,64^. To ensure consistency across datasets, we resample the Sentinel-2 bands to a 30 m by 30 m pixel size using the cubic resampling method, aligning its spatial resolution with that of Landsat. This resampling ensures that the Landsat and Sentinel-2 pixel-pairs are perfectly aligned and cover the same ground target. Utilizing the SDC infrastructure, we combined observations from Landsat-5 and Landsat-7 (July 1999 to May 2005) and Landsat-8 and Sentinel-2 (July 2015 to December 2021), with single satellite observations from Landsat-5 and Landsat-8 during December 1984 to July 1999 and June 2003 to June 2015, respectively. By considering the maximum NDSI value per pixel per month, we generated multi-sensor monthly NDSI products for 1984 to 2021, increasing the average cloud-free data per monthly product from 60% to 65%.

### Step 3 – Summer snow cover mask

The third step is performed on the binary snow cover maps (after processing through the GLMM model). We assume that if a pixel is classified as having snow presence during summer, it will also display snow presence in other seasons. Based on this, we created a summer snow cover mask (SSCM) by calculating the minimum monthly NDSI for June, July, and August over 36 years, resulting in 108 minimum NDSI products. We then computed a summer mean NDSI product using pixel-wise averages for pixels with over 80% cloud-free data during the study period. This result was passed through the GLMM to obtain a minimum SSCM. Pixels within the SSCM classified as clouds from September to May were reclassified as snow. The SSCM consists of 87,122 snow pixels, representing 0.21% of Switzerland’s coverage, and removing approximately 0.14% of cloudy pixels.

### Step 4 - Monthly probability fields

The monthly probability fields step, adapted from Gafurov and Bardossy^31^ utilizes the observation that certain pixels in Switzerland consistently exhibit snow presence or absence during specific months. This approach uses the entire monthly snow cover time series products to detect persistent spatial patterns of snow or land cover for each month. For each pixel, a monthly probability is calculated, representing the likelihood of being snow-covered or snow-free during a specific month. Only pixels with more than 80% cloud-free data over the entire period are included in the calculation of the monthly probability fields. This results in twelve probability maps (on for each month) for Switzerland.

To quantify the predictive power by pixel, two indexes are used: the snow predictability index (SPI) and the land predictability index (LPI). These indexes measure the fraction of snow or land pixels for which the probability of being snow-covered or land is 1 for a given month. To account for climatic variability, a buffer is introduced, representing a vertical elevation shift from the month-specific minimum snow line and maximum land line. This buffer is based on the maximum observed variation in the equilibrium line altitude (ELA) of the Claridenfirn glacier, providing a conservative estimate to account for potential variations in the snow line within the study area. Using these monthly probability fields, cloud pixels are reclassified: as snow if they are above the minimum snow line plus the buffer, and as land if they are below the maximum land line minus the buffer. This step reduces cloud pixels by 6.9% and is particularly effective in summer, reducing cloud cover by 19.4%.

### Step 5 - Snow line

The snow line approach, inspired by the methodologies of Gafurov and Bardossy^31^ and Parajka *et al*.^32^, relies on the topographical elevation of snow and land pixels. It identifies the minimum elevation where snow is present and the elevation above which all pixels are consistently snow-covered, called as the “snow line”. To determine snow line elevations, only snow cover products with at least 60% clear pixels in the study area and a 1% snow-pixel threshold^24^ were used. Following this, the elevation band identified as the minimum snow altitude must contain at least 10% of clear data (snow and land) and 1% of snow pixels^65,66^. Out of 429 snow cover products analysed, 273 (63%) met these requirements to pass the snow line step. This step was relatively effective, reducing cloud cover by an average of 9.6% across the analysed snow cover products.

### Step 6 – Spatial gap-filling: majority filter

The sixth step, based on Gafurov and Bardossy^31^, involves spatially combining neighboring pixels. Each cloudy pixel was reassigned based on the majority value of its four adjacent neighbors. Specifically, if at least three neighboring pixels were identified as snow-covered, the cloudy pixel was reclassified as “snow”. The same principle applied to land-covered pixels. While this assumption may not always be accurate, the likelihood of the middle pixel having the same coverage as its neighboring pixels was generally higher than the opposite scenario.

### Step 7 – Spatial gap-filling: elevation filter

In the final step, alongside the majority filter, we implemented an elevation filter based on the methodology by Gafurov and Bardossy^31^. This step involved evaluating both elevation and land cover of the eight neighboring pixels. If any neighboring pixel was identified as snow-covered and had a lower elevation than the cloudy pixel, the cloudy pixel was reclassified as “snow”. This approach is grounded in the principle that temperature generally decreases with increasing elevation, suggesting that snow persists longer at higher elevations than at lower ones. Therefore, if a pixel is classified as snow and any neighboring pixel at a higher elevation is obscured by clouds, it is reasonable to infer that the higher elevation should also be covered by snow. Although these final two gap-filling steps removed only 1.6% and 0.6% of clouds respectively, they were included due to their low error rates.

Table 1 presents the cloud cover fraction derived from the raw NDSI dataset (independent of the sensors used) and the cloud cover fraction after each step of the SOfS algorithm, both for the entire dataset and by season. This fraction represents the average of the monthly products over the period from 1984 to 2021. The performance of each step is quantified by the fraction of clouds removed during that step, relative to the total number of clouds removed across all steps. It can be observed that the effectiveness of each step varies depending on the season.

**Table 1:** Cloud fraction (%) of the raw NDSI datasets and after implementation of each gap filling step, both for the entire dataset and by season.

	Raw data	Step1	Step2	Step3	Step4	Step5	Step6	Step7	Total
**Fraction of cloud (%)**	57.9	40.0	36.0	35.9	33.5	30.2	29.8	29.6	−48.9
**Fraction of cloud (%) by season**									
**DJF**	59.0	47.0	43.0	42.9	42.4	40.8	39.7	39.4	−33.2
**MAM**	57.7	40.5	36.1	36.0	34.2	31.1	30.7	30.4	−47.3
**JJA**	54.6	31.5	28.6	28.6	23.1	17.8	17.7	17.7	−67.7
**SON**	61.3	41.1	36.4	36.4	34.5	31.5	31.2	31.0	−49.4
**Performance of each step (%)**	—	63	14	0	9	11	2	1	100
**Performance of each step (%) by season**									
**DJF**	—	61	20	0	2	8	6	1	100
**MAM**	—	63	16	0	7	11	2	1	100
**JJA**	—	63	8	0	15	14	0	0	100
**SON**	—	67	16	0	6	10	1	0	100

The performance expressed in % indicates the fraction of clouds removed in each step divided by the total number of clouds removed overall (across all steps). The “Total” column shows the overall reduction of cloud cover fraction.

#### Validation

The accuracy of the produced satellite EO derived snow cover maps are then evaluated using a confusion matrix using *in-situ* measurements provided by MeteoSwiss as well as various statistical metrics such as number of true positive, false positive, false negative, and true negative; overall accuracy (OA), the TSS, sensitivity and specificity indices at all stations. These methods are further detailed in the Technical Validation section.

#### Data publication

Finally, to ensure that this snow cover data product can be widely and openly accessible to the largest audience possible, all data are published in FAIR national environmental data repository for EO Open Science. This will ensure that the published data are fully compliant with the Findable-Accessible-Interoperable-Reusable (FAIR) principles while at the same time compliant with the widely adopted geospatial standards made available in Spatial Data Infrastructures (SDI), such as OGC Web Map Service (WMS), Web Coverage Service (WCS), Catalog Service for the Web (CSW) and ISO19115/19139 standards^67^.

## Data Records

The final dataset includes 429 monthly files covering the period from December 1984 to December 2021. Certain months are missing, particularly during 2012, due to transitions between satellite missions: landsat-5 ended in November 2011, and landsat-8 began in March 2013. The files are provided in GeoTiff format, using the EPSG:4326 Coordinate Reference System (CRS) and a spatial resolution of 30 m. Table 2 presents the descriptive characteristics of the SOfS-derived snow cover products. Each pixel in the monthly snow cover products is classified with values ranging from 0 to 2, as follows:

- 0 when the pixel is snow-free (i.e., land),
- 1 when the pixel is covered with snow,
- 2 when the pixel is covered with clouds (including cloud shadow),
- Na when the pixel is classified as water or lies outside of Switzerland.

**Table 2:** Descriptive characteristics of the SOfS-derived snow cover dataset.

SOfS-derived data products	Monthly snow cover products
**Datatype**	Raster
**Data Format**	GeoTIFF
**Coordinate Reference System**	EPSG:4326
**Spatial Coverage**	Switzerland
**Temporal Coverage**	December 1984 to December 2021
**Spatial Resolution**	30 m
**Temporal Resolution**	Monthly
**Missing values (yyyy-mm)**	1987-01; 2000-12; all 2012; 2013-01; 2013-02

A static copy of each collection is made available at the University of Geneva Research Data repository (https://yareta.unige.ch)^68^: 10.26037/yareta:nq6phdtx45cz5goqqxfr5qjaei.

The dataset is described with standardized metadata following the ISO19115 schema and description is available at: https://geonetwork.swissdatacube.org/geonetwork/srv/eng/catalog.search#/metadata/d98b8938-22fb-450e-ae1d-af078b6307c8.

In addition, metadata and data are available as Open Geospatial Consortium (OGC) standards web services endpoints to ensure interoperable discovery, visualization and download.

- Metadata Discovery-Catalog Service for the Web (CSW 2.0.2): https://geonetwork.swissdatacube.org/geonetwork/srv/eng/csw?request=GetCapabilities&service=CSW&version=2.0.2.
- Data visualization -Web Map Service (WMS 1.3.0): https://ows.swissdatacube.org/wms?request=GetCapabilities&service=WMS&version=1.3.0 and Web Map Tile Service (WMTS 1.0.0): https://ows.swissdatacube.org/wmts?request=GetCapabilities&service=WMTS&version=1.0.0.
- Data download–Web Coverage Service (WCS 1.0.0,2.0.0,2.1.0): https://ows.swissdatacube.org/wcs?request=GetCapabilities&service=WCS&version=2.1.0.

These endpoints enable users to access and/or integrate these datasets in their desktop, web-based clients or own specific analysis workflows.

## Technical Validation

To ensure proper documentation and standardization, SOfS data products were reviewed by a data curator prior to being uploaded to SwissEnvEO. This review included metadata verification and compliance with standardized formats, such as naming conventions, reference systems, spatial resolution, and geographic extent. These satellite-derived snow products underwent rigorous statistical validation to assess their accuracy and reliability.

**Fig. 3:**
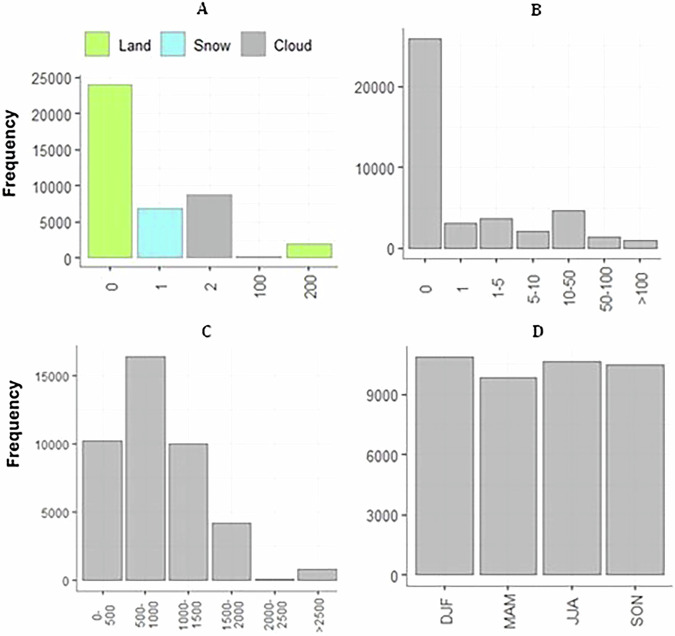
Frequency distribution of four variables (snow cover products observations (**A**), snow depth (**B**), elevation (**C**), and seasons (**D**)) for the snow cover products and *in-situ* measurements matchups at 263 stations for the period 1984 to 2021 in Switzerland.

The validation process involved constructing a confusion matrix by comparing 33,026 pairs of cloud-free satellite-based snow observations (SOfS-derived dataset) with *in-situ* snow depth measurements from 264 climate stations distributed across Switzerland. These *in-situ* measurements, considered as ground truth, were obtained from the Federal Office of Meteorology and Climatology (MeteoSwiss) archive portal (https://gate.meteoswiss.ch/idaweb). The *in-situ* dataset spans a wide range of elevations from 225 to 2540 m a.s.l. and includes monthly average of snow depth measurements for each station, calculated using the specific days when satellite scenes were acquired to ensure temporal alignment with the SOfS-derived snow cover products. The validation period extends from December 1984 to December. Snow depth observations from the climate stations were spatially matched to overlaying satellite pixels, resulting in 33,026 cloud-free *in-situ*-satellite matchups. Figure 3 presents the frequency distribution of key variables in the validation dataset, including snow cover observations, snow depth, elevation, and seasonality. The agreement between *in-situ* snow observations and remote sensing data was evaluated for two snow depth thresholds (SDthr): 1 cm and 5 cm. These thresholds were chosen based on their significance in determining the concordance between ground-based and remote sensing snow observations, as identified in previous studies^65,69,70^. Shallow snow depths (<1 cm) are particularly known to diminish the consistency in snow-related analyses^71^. The confusion matrix allowed us to identify commission errors in the SOfS products (i.e., false positive, where SOfS indicates snow but *in-situ* data indicates no snow), omission errors (i.e., false negative, where SOfS indicates no snow but *in-situ* data indicates snow), and agreements (where both SOfS and *In-Situ* data agree on snow or no snow).

Additionally, we calculated the OA^72^ metric to quantify the agreement between the SOfS-derived monthly snow cover products and the mean monthly snow depth measurements from *in-situ* stations over the study period. The OA is calculated as follows:

$${OA}\left( \% \right)=\frac{{true\; positive}+{true\; negative}}{{true\; positive}+{false\; postive}+{false\; negative}+{true\; negative}}\ast 100$$

The OA metric provides insight into the proportion of *in-situ* measurements that were correctly identified in the SOfS products.

Finally, the TSS^73^ was applied to evaluate the agreement between snow depth observations at ground stations and the monthly SOfS products. The TSS incorporates sensitivity and specificity, offering a robust measure of overall agreement.

$${TSS}\left( \% \right)=\frac{{\rm{ad}}-{\rm{bc}}}{({\rm{a}}1{\rm{c}})({\rm{d}}+{\rm{b}})}\ast 100=\left({Sensitivity}+{Specificity}-1\right)\ast 100$$

$${Sensitivity}=\frac{{\rm{a}}}{{\rm{a}}+{\rm{c}}}$$

$${Specificity}=\frac{{\rm{d}}}{{\rm{d}}+{\rm{b}}}$$

Where a, b, c and d represent respectively the number of true positives, false positives, false negatives, and true negatives. Based on Cohen’s Kappa index, TSS is a threshold-dependent evaluation metric ranging from -1 to 1, with 1 indicating perfect agreement and values ≤ 0 signifying performance no better than random classification^72^. TSS values > 0.75 are considered excellent, 0.40–0.75 are good, and <0.40 are poor. TSS serves as an alternative accuracy measure, addressing Kappa’s prevalence dependence while retaining its benefits^72^. Sensitivity measures the proportion of observed snow presence correctly predicted by remote sensing, while specificity measures the proportion of observed snow absence correctly predicted.

The confusion matrix and corresponding metrics (Table 3) indicate excellent agreement between the SOfS dataset and *in-situ* snow depth measurements, with overall accuracy ranging from 91% (SDthr = 1 cm) to 93% (SDthr = 5 cm). SOfS products exhibit superior performance in classifying snow-free areas compared to snow-covered ones, with a slight tendency to underestimate snow cover. For SDthr = 1 cm, 73% of 9,054 snow-covered days were correctly classified, while 27% were misclassified as snow-free. For 23,972 snow-free days, 98% were correctly classified, with only 2% misclassified. The overall accuracy increases with SDthr, but the false negative rate (3%/6%) doubles at 5 cm.

**Table 3:** Confusion matrix and associated metrics are computed for SOfS products and *in-situ* snow depth measurements at 264 climate stations.

(SDthr = 1/5)		Snow (1)	No Snow (0)
***In-situ***	**Snow (SD ≥ SDthr)**	6586/5490	2468/783
	**No snow (SD ≤ SDthr)**	499/1595	23473/25158
		**All**	
**Overall accuracy OA (%)**		91/93	
**Sensitivity (%)**		73/87	
**Specificity (%)**		98/94	
**TSS (%)**		71/81	

This analysis is performed specifically for cloud-free pixel for the period 1984–2021, considering two different snow de depth thresholds (SDthr). The statistical analysis is conducted for all *in-situ-*satellite matchups (All).

TSS analysis shows good agreement (71%) for SDthr = 1 cm and excellent agreement (81%) for SDthr = 5 cm. Specificity (98%-94%) is higher than sensitivity (71%-87%), indicating a higher likelihood of omitting snow-covered pixels than misclassifying snow-free ones. Specificity decreases with increasing SDthr. Gap-filling procedure accuracy ranges from 95% (SDthr = 1 cm) to 97% (SDthr = 5 cm), with lower sensitivity than specificity at 1 cm, indicating underestimation of snow cover.

Seasonal analysis reveals variability in classification accuracy. Most false negatives occur in winter (DJF, 33%) and spring (MAM, 34%), with only 8% in summer (JJA). False positives are more frequent in winter (53%) but absent in summer. Overall, summer shows the highest AO (90%) with perfect specificity (100%). In contrast, winter exhibits the lowest OA (82%) due to reduced specificity, reflecting the challenges posed by frequent snowfall events and variable snow depths. Autumn and spring have 87% OA, with lower sensitivity compared to specificity. Analysis by altitude bands (500 m intervals) shows consistently high OA (>84%, regardless of SDthr) across most elevation, except for the 2000–2500 m range, where OA drops slightly. This transition zone accounts for only 0.1% of the validation dataset, which may contribute to the lower performance observed. The individual performance of the gap-filling procedure, starting from step 2, was assessed by evaluating its capacity to correctly classify cloud-covered pixels as either snow-covered or snow-free (i.e., land). The OA for gap-filling ranges from 95% (SDthr = 1 cm) to 97% (SDthr = 5 cm). However, as observed for the entire dataset, sensitivity was notably lower than specificity at SDthr = 1 cm, confirming that the procedure tends to underestimate snow cover.

## Usage Notes

The snow cover dataset derived from the SOfS algorithm provides a long-term, spatially explicit record of snow cover dynamics at medium-to-high resolution. It complements existing *in-situ* snow observations and serves as a valuable resource for monitoring snow cover changes over Switzerland from 1984 to 2021. This dataset is suitable for applications such as assessing snow cover extent over regions, analyzing pixel-based temporal changes, and tracking snowline evolution. Researchers can use it at various spatial scales (e.g., watersheds, biogeographic regions, or administrative units), making it adaptable for hydrological, climatic, and environmental studies.

Key limitations include the dataset’s monthly temporal resolution, which may not capture short-term dynamics such as rapid snowmelt or snowfall events. Additionally, despite a gap-filling procedure, a residual cloud fraction of ~29% remains, which may affect the accuracy of snow cover extent measurements in certain regions or periods.

With its 37-year temporal span, the dataset is well-suited for integration into climate models and other analytical frameworks, offering valuable insights into long-term snow cover trends and dynamics.

## Data Availability

The SDC is based on the Open Data Cube platform that can be obtained at: https://www.opendatacube.org and freely available under an Apache 2.0 license (https://opensource.org/licenses/Apache-2.0). JupyterLab can be freely obtained at: https://github.com/jupyterlab/jupyterlab under a 3-clause BSD license (https://opensource.org/license/bsd-3-clause). The Snow Observations from Space algorithm is available as Jupyter notebooks at: https://github.com/poussinc/SOfS-algorithm and freely available under an Apache 2.0 license (https://opensource.org/licenses/Apache-2.0).
